# Galvanic Vestibular Stimulation Produces Cross-Modal Improvements in Visual Thresholds

**DOI:** 10.3389/fnins.2021.640984

**Published:** 2021-03-31

**Authors:** Jamie L. Voros, Sage O. Sherman, Rachel Rise, Alexander Kryuchkov, Ponder Stine, Allison P. Anderson, Torin K. Clark

**Affiliations:** Ann & H.J. Smead Department of Aerospace Engineering Sciences, University of Colorado-Boulder, Boulder, CO, United States

**Keywords:** GVS, stochastic resonance (SR), cross-modal, in-channel, visual thresholds, white noise

## Abstract

**Background:**

Stochastic resonance (SR) refers to a faint signal being enhanced with the addition of white noise. Previous studies have found that vestibular perceptual thresholds are lowered with noisy galvanic vestibular stimulation (i.e., “in-channel” SR). Auditory white noise has been shown to improve tactile and visual thresholds, suggesting “cross-modal” SR.

**Objective:**

We investigated galvanic vestibular white noise (nGVS) (*n* = 9 subjects) to determine the cross-modal effects on visual and auditory thresholds.

**Methods:**

We measured auditory and visual perceptual thresholds of human subjects across a swath of different nGVS levels in order to determine if some individual-subject determined best nGVS level elicited a reduction in thresholds as compared the no noise condition (sham).

**Results:**

We found improvement in visual thresholds (by an average of 18%, *p* = 0.014). Subjects with higher (worse) visual thresholds with no stimulation (sham) improved more than those with lower thresholds (*p* = 0.04). Auditory thresholds were unchanged by vestibular stimulation.

**Conclusion:**

These results are the first demonstration of cross-modal improvement with galvanic vestibular stimulation, indicating galvanic vestibular white noise can produce cross-modal improvements in some sensory channels, but not all.

## Highlights

- White noise as applied to the vestibular system (nGVS) results in a reduction of visual perceptual thresholds.
- Reduction is visual perceptual thresholds is negatively correlated with initial visual perceptual threshold (those with higher thresholds to begin with stand to gain the greatest improvement).
- No such reduction was seen in auditory thresholds with the application of nGVS.

## Introduction

Stochastic resonance (SR) is a phenomenon whereby an input signal to a non-linear system is enhanced by the presence of a particular non-zero level of noise (Gammaitoni et al., 1989). SR in physiological sensory systems has been observed, in which a faint signal is perceived more easily with the addition of white noise (Gammaitoni et al., 1989; Wiesenfeld and Moss, 1995; Moss et al., 2004; McDonnell and Abbott, 2009). In-channel SR refers to stochastic resonance occurring within the same sensory modality (e.g., auditory white noise improving auditory perception. Cross-modal SR refers to stochastic resonance occurring outside the sensory modality of the white noise (e.g., vestibular white noise improving visual perception).

**FIGURE 1:**
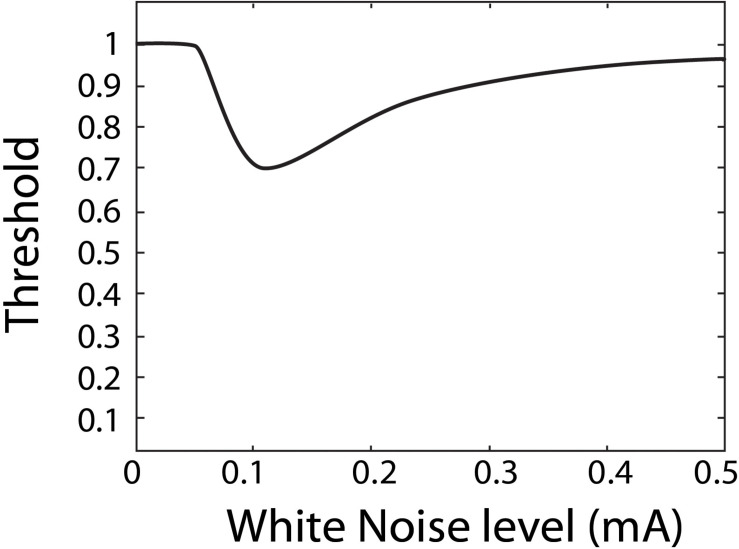
Graph to show characteristic shape of SR the curve in threshold against noise level.

Stochastic resonance has often been investigated and observed through psychophysiological experiments, aimed at quantifying perceptual thresholds (Zeng et al., 2000; Manjarrez et al., 2007; Ries, 2007; Lugo et al., 2008; Galvan-Garza et al., 2018; Keywan et al., 2018). A perceptual threshold is the smallest stimulus input that can still be reliably perceived by a person. For example, an auditory threshold refers to the faintest sound one can still reliably hear. In the domain of perceptual thresholds, SR is thought to show a characteristic U-shape of as a function of white noise as shown in Figure 1 (Morse and Evans, 1996; Moss et al., 2004; Lugo et al., 2008; McDonnell and Abbott, 2009). Specifically, as more white noise is added it is thought to resonant with the stimulus to produce a reduced perceptual threshold, but once too much white noise is added it is no longer beneficial, and for some in-channel sensing modalities can degrade perception. SR in the visual channel is a well-documented occurrence in subjects with healthy vision (Riani and Simonotto, 1994; Simonotto et al., 1997, 1999; Piana et al., 2000) and has also been demonstrated in visually impaired subjects (Itzcovich et al., 2017). Auditory white noise has been shown to lower auditory thresholds in subjects with healthy hearing (Zeng et al., 2000; Ries, 2007; Sherman, 2019) and those with cochlear implants (Zeng et al., 2000). Tactile white noise has been found to improve touch (Collins et al., 1996, 1997; Richardson et al., 1998; Enders et al., 2013) and vestibular white noise to improve perceptual thresholds (Mulavara et al., 2011, 2015; Samoudi et al., 2014; Galvan-Garza et al., 2018; Keywan et al., 2018, 2020; Wuehr et al., 2018) as well as associated vestibular responses such as balance in the dark, spinal reflexes, and locomotion (Mulavara et al., 2011, 2015; Samoudi et al., 2014; Wuehr et al., 2018; Keywan et al., 2020).

Vestibular perception may be altered by applying electrical white noise via electrodes placed on the mastoids, referred to as galvanic vestibular stimulation (GVS; Goel et al., 2015; Galvan-Garza et al., 2018; Keywan et al., 2018; Wuehr et al., 2018). Improvements in roll tilt vestibular thresholds exist within the subject pool but are not consistent from subject to subject, ranging from a 50% reduction in threshold to no improvement at all (Galvan-Garza et al., 2018; Keywan et al., 2018). There are also inconsistencies as to the electric current level of noisy GVS (nGVS) eliciting an improvement in vestibular thresholds (Galvan-Garza et al., 2018; Keywan et al., 2018). Vestibular stimulation in healthy subjects appears to only produce benefits during active stimulation (Keywan et al., 2020), while others have suggested improved balance in elderly patients even after stimulation has ceased (Fujimoto et al., 2016).

Cross-modal SR is achieved when improvements in perception occur in a different channel from that of the white noise stimulation (Manjarrez et al., 2007; Lugo et al., 2008). Previous studies have suggested that applying auditory white noise can improve visual flicker sensitivity (Harper, 1979; Manjarrez et al., 2007), visual contrast thresholds (Lugo et al., 2008) and motor control (Ai et al., 2009). We note relevant caveats to these studies: The first study (Manjarrez et al., 2007) statistically compares sham thresholds to each individual subject’s best threshold (which do not all occur at the same noise level) without any re-measuring, producing a biased sample and an increased likelihood of a false positive. The third (Lugo et al., 2008) does not statistically assess findings, but rather demonstrates descriptive improvements. The second and fourth (Harper, 1979; Ai et al., 2009) use data from just three and four subjects, respectively. All studies support the notion that there is not one white noise level that is optimal for all subjects, as in all studies (Harper, 1979; Manjarrez et al., 2007; Lugo et al., 2008; Ai et al., 2009) each subject had an individually determined optimal stimulation level. Another study showed tactile stimulation to enhance speech recognition in subjects with cochlear ear implants (Huang et al., 2017), the cause was later hypothesized to be due to the multisensory nature of the dorsal cochlear nucleus (Krauss et al., 2018). Lugo et al. (2008), measured thresholds using a condition in which auditory white noise was applied, as well as with a 3D like sound designed to control for stimulating attention (but that would presumably not induce SR). They found an improvement in tactile thresholds only with white noise stimulation (not the 3D like sound) and concluded that the reduction in thresholds was not due to attention or arousal effects (Lugo et al., 2008). We are not aware of any other studies investigating cross-modal SR nor are we aware of any studies investigating cross-modal SR using white noise GVS.

Here, we aimed to test for the presence of cross-modal SR in auditory and visual sensory modalities with the application of nGVS. We built upon observations of in-channel SR in auditory and visual modalities and the previously investigated cross-modal benefits of auditory white noise. Instead of auditory white noise, we explored using GVS owing to its efficacy in improving vestibular thresholds and balance. Since many studies have demonstrated optimal noise levels to achieve SR are individualized (Manjarrez et al., 2007; Lugo et al., 2008; Galvan-Garza et al., 2018), our methods ensure independent samples between thresholds measured with nGVS and thresholds measured without nGVS (sham). By first determining the best nGVS level (for each subject), we were able to then re-measure the subjects’ threshold with no stimulation (sham) and with nGVS for two independent, randomized samples for a paired statistical test.

## Materials and Methods

### Subjects

Ten unique subjects were enrolled and passed the screening criteria described below (4F, ages 18–25 mean 21.4 years). Nine subjects completed all testing for both visual and auditory threshold tasks, one subject completed only the visual task and one other subject did not do the re-measure (see SR detection) protocol in the visual task.

Sample size was estimated based upon a power analysis with the power set to β = 0.80 and the error rate to α = 0.05. Based upon previous studies, we anticipated a 30% improvement in visual thresholds (Lugo et al., 2008; Galvan-Garza et al., 2018) with nGVS applied. Using simulations we have published previously (Voros et al., 2020), we expected measurement uncertainty (coefficient of variation) for the difference in thresholds between sham and the best nGVS level to be 0.2. This analysis suggested five subjects would be required, motivating us to conservatively aim for 10 subjects.

During pre-screening, all subjects self-reported no known history of vestibular dysfunction, tactile dysfunction, auditory dysfunction, or vision that could not be corrected with contact lenses. During screening, we verified that subjects did not have excessive earwax and outer ear damage through an otoscopic examination. Next, we completed a tympanogram to verify subjects had no active or recent middle ear pathology by confirming they had normal tympanometry (defined as peak pressure between −100 and +50 daPa, canal volume between 0.6 and 1.9 ml, and static admittance between 0.3 and 1.7 ml). Finally, all subjects had normal hearing, or baseline audiometric thresholds ≤25 dB HL up to 8 kHz, as determined through a pure-tone Békésy-style tracking procedure. Three potential subjects were removed due to requiring glasses (and not contact lenses) in order to have normal vision, which were not compatible with our testing apparatus. No subjects had lesions anywhere their skin came into contact with any testing equipment and no subjects self-reported electronic implants in the head. All procedures were approved by the University of Colorado-Boulder Institutional Review Board, conducted in accordance with the Declaration of Helsinki and all subjects provided written informed consent.

### Study Design

After screening, subjects returned to the laboratory on two subsequent visits (separate days within a two-week period) to complete testing. One visit tested all visual thresholds and the other all auditory thresholds, with the ordering randomized. The GVS electrodes were (re)applied and removed at the beginning and end of each testing visit.

The GVS system was donned prior to any testing and worn for the entirety of testing (including during sham condition), however, stimulation was only applied during threshold measurement sessions. Subjects were not informed of how or when they would receive GVS or what sensations it might produce. Subjects were provided a several minute break between sessions, but the electrodes were not removed. Galvanic vestibular white noise was applied bilaterally via electrodes placed on the mastoids. Broadband (0–100kHz), unipolar, zero-mean white noise was generated by the stimulator (Soterix Medical Inc., Model 0810) and delivered via leads connected to electrodes with a total contact area of 2 cm^2^. The surface of the skin was prepared with Nuprep skin prep gel and cleaned with alcohol wipes. Electrodes were then placed, secured with a headband, and then Signagel electrode gel (Parker Labs) was injected to the electrode sites. Stimulation was applied only after impedance was indicated as acceptably low by an indicator on the device. The magnitude level of the white noise stimulation was defined as the peak current level.

Thresholds (either visual or auditory, see section “Perceptual Thresholds”) were assessed over a range of nGVS current levels from 0 to 1 mA in increments of 0.1 mA in a randomized order. The subject-specific nGVS level which yielded the best perception (i.e., their “best” nGVS level or bnGVS) was defined as the white noise level (not including sham) resulting in the lowest measured threshold. The subjects’ perceptual thresholds at the sham and bnGVS noise levels were then re-measured to generate independent samples. The order in which the re-measured sham threshold and threshold at bnGVS level were tested was also randomized. The bnGVS level was determined independently for auditory and visual thresholds, such that a given subject often had different bnGVS levels for the two threshold modalities.

All threshold measurements were performed inside a darkroom and sound booth to minimize sensory cues outside the modality in which the threshold was being measured. Subjects and test operators were blinded to the stimulation condition. It is possible that at the highest stimulation levels some subjects could have felt a tingling sensation, but they were not primed to know this would have meant higher levels of GVS stimulation.

### Perceptual Thresholds

Thresholds were measured with a two-alternative forced-choice detection task, in which that subject had to identify which of two sequential intervals the stimulus was in. The stimulus (e.g., auditory tone) always occurred in either the first or second interval, with no stimulus (e.g., no auditory tone) occurring in the other, determined randomly for each trial. Subjects responded verbally (e.g., “interval one” or “interval two”) to indicate which interval they thought contained the stimulus. An adaptive 3 down 1 up Parametric Estimation by Sequential Testing (PEST; Taylor and Creelman, 1967; Leek, 2001; Karmali et al., 2016) procedure was used to determine the magnitude of the stimuli (e.g., loudness of the auditory tone) for each trial. Subject responses were fit with a cumulative Gaussian psychometric function (Green and Swets, 2000; Merfeld, 2011; Karmali et al., 2016) scaled from 0.5 to 1 (since guessing performance would yield 0.5% correct with the two alternatives). The cumulative Gaussian was parameterized by two values, μ and σ. The μ value (of a cumulative Gaussian fit to trials from a two-interval task) represented the stimulus level at which the subject stands to get 75% of trials correct, which we defined as the threshold.

The threshold estimation theoretically becomes more precise with more trials to which the psychometric curve can be fit. However, subject fatigue, focus, and availability can practically constrain this benefit. Informed by performing Monte-Carlo simulations (Voros et al., 2020) alongside pilot studies, we chose to perform 50 trials for each visual threshold test (at a given white noise level) and 100 trials for each auditory threshold test. Similarly, re-measures had 50 trials at each of sham and bnGVS for visual thresholds and 100 for auditory.

**FIGURE 2:**
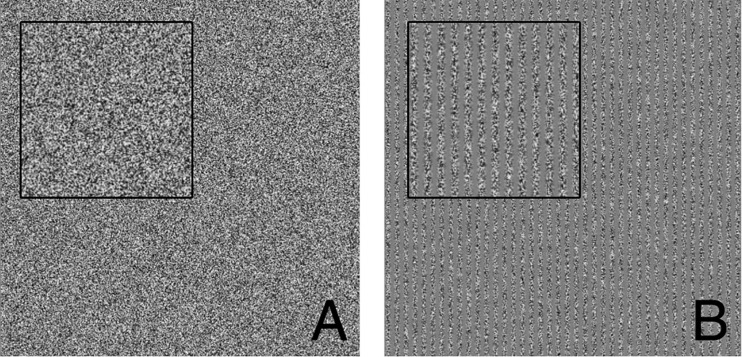
Visual threshold task example presentations. **(A)** Patch containing only visual static noise (i.e., no signal). **(B)** Patch containing 40 vertical gratings (i.e., signal). Insets each show close ups of visual stimuli to make it more apparent that panel **(B)** contains vertical gratings. Subjects were tasked with determining which interval presentation (first or second) contained the vertical gratings.

We used contrast gratings to measure visual contrast thresholds (Foley et al., 2007). In each 1 second interval, subjects were presented with one of the types of patches shown in Figure 2. Subjects had to identify which interval contained the patch with the grating. Each visual grating (Figure 2) was 21 cm tall and wide (square) and was presented on an otherwise gray computer monitor placed 105 cm in front of the seated subject near eye level.

Auditory thresholds were measured in the right ear with a 1 kHz pure tone stimulus of 0.5 s in duration. Subjects were presented with two 0.5 s intervals sequentially in which one (and only one) interval contained the auditory tone. Subjects had to identify which interval contained the tone. Auditory tones were administered via a device (Creare Hearing Assessment, Creare Inc.) and though over-the-ear headphones. We chose a shorter stimulus for the auditory task in order to compensate for the larger number of trials in an effort to keep the session durations manageable and minimize the potential for fatigue (i.e., 0.5 s tones with 100 trials took roughly equal time to 1 s visual stimuli and 50 trials).

### Analysis

A two tailed *t*-test was performed between the re-measured sham thresholds and re-measured thresholds with stimulation. The Shapiro–Wilk test for normality was performed on the paired differences to ensure normal distribution of visual and auditory thresholds.

**FIGURE 3:**
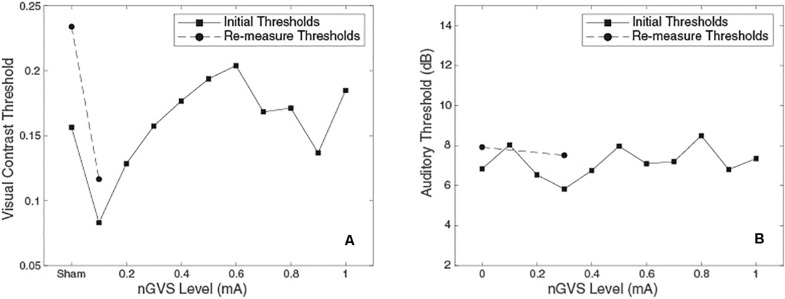
Plots of threshold against nGVS level for one example subject. **(Left)** Visual contrast threshold measurements, bnGVS is at 0.1 mA. **(Right)** Auditory threshold measurements, bnGVS is at 0.3 mA.

In order to detect the characteristic U-shape associated with SR, we used a subjective human classification method previously described (Chaudhuri and Merfeld, 2013; Karmali et al., 2016; Galvan-Garza et al., 2018; Voros et al., 2020). Briefly, judges viewed plots of measured threshold versus nGVS level, similar to those shown in Figure 3A. Judges were given plots of both simulated and actual subject data and were asked to classify each plot as exhibiting SR or not exhibiting SR (expected no U dip). Plots of experimental subject data were randomly interspersed with plots from simulated subjects. Simulated subjects were modeled with the same experimental protocol of real subjects (e.g., number of trials, adaptive sampling, psychometric curve fitting) (Chaudhuri and Merfeld, 2013; Karmali et al., 2016; Galvan-Garza et al., 2018; Voros et al., 2020). Critically, the measured thresholds include measurement variability due to the finite number of trials, such that classifying each plot as exhibiting SR was non-trivial (as it is with experimental subject data). Modeled subjects exhibited a 50% split of exhibiting SR or not. In the simulated subjects with underlying SR, we assumed an underlying threshold reduction of 30% at the minimum of the U-shape, motivated by that previously observed (Galvan-Garza et al., 2018). Two human judges classified 90 simulated subjects along with 10 subjects for visual thresholds and nine subjects for auditory thresholds (recall that of the 10 subjects who completed the visual thresholds, one did not return to complete the auditory thresholds). Both judges are authors and are familiar with SR curve shape, but they were blinded as to whether each plot was simulated or an experimental subject. While classifications were subjective (Chaudhuri and Merfeld, 2013; Karmali et al., 2016; Galvan-Garza et al., 2018; Voros et al., 2020), this previously established approach assessed whether subject’s exhibited the characteristic SR curve, while controlling for false positives using simulations either with or without underlying SR. Classifications were assessed via chi-squared tests to test for differences in classification between three groups: simulated subjects with SR, simulated subjects without SR and actual subjects. Chi-squared test were performed on two groups at a time. For example, a chi-squared test between actual subject classifications and simulated subjects with SR classifications can indicate if the proportion of plots the judges classified as having SR differed between the two groups.

## Results

Figure 3 shows an example subject’s data. For the visual thresholds (Figure 3A) with nGVS of 0.1 mA the threshold is reduced (i.e., improved) relative to the sham threshold. Further increases of nGVS cause the thresholds to increase to near or above the sham threshold. The re-measure thresholds (shown as circles) performed at the bnGVS level of 0.1 mA and sham, also show a lower threshold at 0.1 mA as compared to sham. The auditory thresholds for this same subject (Figure 3B) are fairly consistent for each level of nGVS provided. The bnGVS level is identified as 0.3 mA, but re-measuring the threshold with bnGVS did not yield as large an improvement over the re-measured sham as bnGVS did with visual contrast thresholds. This example subject shows the threshold response versus nGVS levels and the thresholds for the re-measure procedure.

### bnGVS Levels

**FIGURE 4:**
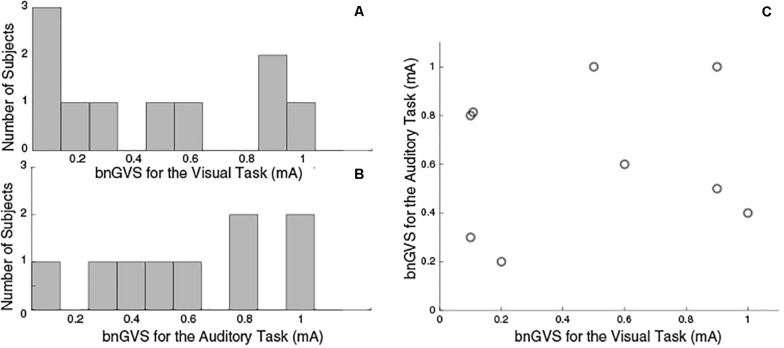
Histograms to show GVS levels resulting in the lowest threshold measurement. **(A)** Visual Task bnGVS. **(B)** Auditory task bnGVS. **(C)** Scatter plot to show individual subjects’ bnGVS level across each task, we see no correlation. The visual task had 10 subjects complete testing and the auditory task had just nine.

Similar to previous studies (Galvan-Garza et al., 2018; Keywan et al., 2018), we see considerable variation across subjects in the nGVS level resulting in the lowest measured threshold (i.e., the best nGVS level, bnGVS). Figure 4 shows histograms of best nGVS level split by task (visual, auditory) because we did not assume that it would be consistent across tasks. The best nGVS level for both tasks varied across the full range we tested, from 0.1 mA to 1 mA in intervals of 0.1 mA. Further Figure 4C shows that the best nGVS level was not consistent for an individual between the visual and auditory tasks.

### Indicators of SR

**FIGURE 5:**
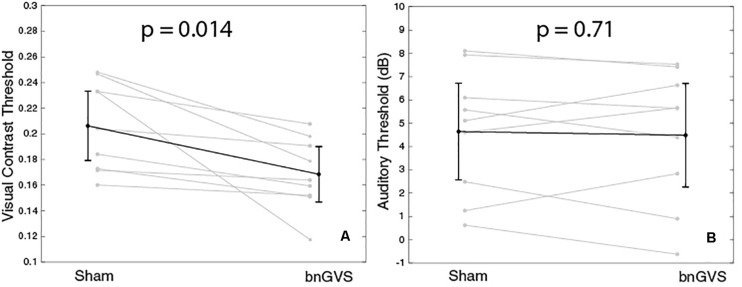
Plots to show visual and auditory thresholds with and without GVS. Panel **(A)** shows a statistically significant improvement in visual contrast thresholds with bnGVS. **(B)** shows small or no change in auditory thresholds.

We performed independent re-measures of the sham and that which was determined to be the best GVS white noise level so that we could test for a difference with and without nGVS. The resulting thresholds for these re-measures are shown in Figure 5 for each of the visual (panel A) and auditory (panel B) tasks.

The visual thresholds were statistically significantly lower in the re-measure with the subject-specific bnGVS than in the re-measure with sham (paired *t*-test, *t*(8) = 3.15, mean difference = −0.038, *p* = 0.014, 95% confidence interval (CI) = [−0.06, −0.01]). All subjects showed an improved threshold with the mean improvement of 0.038 corresponds to an 18% improvement relative to the mean sham threshold.

For the auditory thresholds, there was no significant difference found between the sham and best re-measures (paired *t*-test, t(8) = 0.38, mean difference = −0.16 dB, *p* = 0.71, 95% CI = [−0.8,1.1]). While some subjects did have slight improvements in the re-measure with bnGVS, several subjects actually had worse thresholds with bnGVS.

**FIGURE 6:**
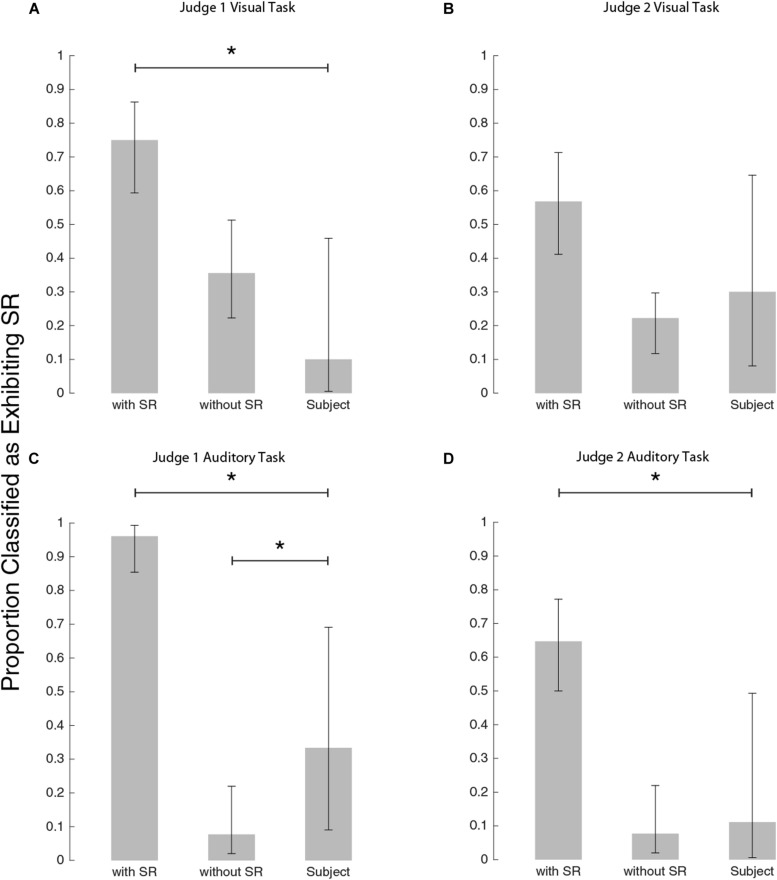
Bar plots to show how the judges classified each group. **(A)** Judge #1 on visual task, **(B)** Judge #2 on visual task, **(C)** Judge #1 on auditory task, and panel **(D)** Judge #2 on auditory task. Stars indicate a significant difference between classification proportions by Chi-square tests (see text for details).

In order to determine whether SR was the underlying mechanism responsible for threshold improvement, as in previous studies (Galvan-Garza et al., 2018; Voros et al., 2020) we had blind judges classify whether plots exhibited SR (for each of auditory and visual thresholds: 90 simulated subjects each with an equal likelihood of having underlying SR versus not, and our experimental subjects). Figure 6 shows the outcome of the judging process to identify the characteristic U-shaped SR curve. While some of our experimental subjects were classified as having SR, most were not (rightmost bar in each panel). This tended to contrast the simulations which had underlying SR, which were predominantly classified (correctly) as exhibiting SR. Critically, the simulated subjects with no underlying SR were occasionally misclassified as having SR (i.e., a false positive). This highlights the importance of comparing experimental subject outcomes to those simulated with no underlying SR to properly account for false positives.

**FIGURE 7:**
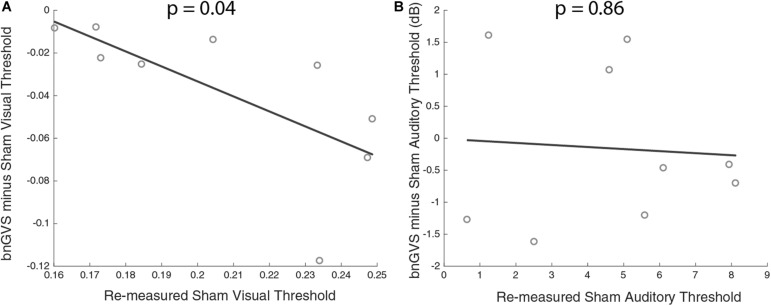
Scatterplots of sham threshold against improvement (negative difference indicating improved threshold) with line of best fit. **(A)** Visual contrast thresholds. **(B)** Auditory thresholds. Correlation between improvement and sham threshold was only found in visual contrast thresholds.

Judge #1 on the visual task (Figure 6A), classified experimental subjects differently from simulated subjects exhibiting SR [χ^2^(DOF = 1, *N* = 54) = 14.8, *p* < 0.001] but not differently from simulated subjects exhibiting no SR [χ^2^(DOF = 1, *N* = 55) = 2.5, *p* = 0.11]. Judge #2 for the visual task (Figure 6B), did not differentiate between simulations with and without SR as well as judge #1. By judge #2’s classifications, the subject pool was not significantly different from either simulation group: simulations with SR as compared to subjects [χ^2^(DOF = 1, *N* = 54) = 2.3, *p* = 0.13] and simulations without SR as compared to subjects [χ^2^(DOF = 1, *N* = 55) = 0.27, *p* = 0.60]. While Judge #2 was inconclusive, Judge #1’s classifications suggest that our subjects’ visual thresholds did not demonstrate the characteristic U-shaped curve associated with the mechanism of SR.

For the auditory task, judge #1’s subject classifications (Figure 6C) were different from both simulations with SR [χ^2^(DOF = 1, *N* = 60) = 26, *p* < 0.001] and without SR [χ^2^(DOF = 1, *N* = 48) = 4.4, *p* = 0.036]. Judge #2’s classifications of subjects were different from simulations with SR [χ^2^(DOF = 1, *N* = 60) = 8.9, *p* = 0.003] and consistent with simulations without SR [χ^2^(DOF = 1, *N* = 48) = 0.11, *p* = 0.74]. While Judge #1’s classifications suggest that the subject pool lies somewhere between simulations with SR and simulations without SR, judge #2’s classifications imply that the subject group is most consistent with simulations without SR. Thus, this blind-judging classification analysis suggests nGVS does not produce the characteristic U-shaped SR curve in either visual or auditory thresholds.

### Relationship Between Sham Threshold and bnGVS Improvement

Next, we examined the relationship between amount of perceptual improvement and sham threshold. Amount of improvement was defined as the difference between sham threshold and the bnGVS stimulated threshold, when re-measured (i.e., negative values correspond to improved thresholds). We found a significant correlation between sham threshold and improvement in visual contrast thresholds only [Pearson correlation *r*(7) = 0.69, *p* = 0.04]. The higher a subject’s sham threshold was, the more that subject stood to benefit from stimulation with their own best nGVS level. Unsurprisingly (since auditory thresholds did not improve with the bnGVS level), no such correlation was found in auditory thresholds [*r*(7) = 0.07, *p* = 0.86].

## Discussion

We have designed and implemented a statistically rigorous method of identifying cross-modal improvements in auditory and visual perceptual thresholds via the use of galvanic vestibular white noise stimulation. Our results demonstrate a statistically significant difference between sham and the subject-specific best nGVS level using independent samples, indicating that the addition of low levels of vestibular white noise elicits improvement in visual contrast thresholds. However, our current methods did not indicate that improvements were consistent with the SR model, as assessed by our human judges. This discrepancy could be because improvements were not due to SR, the SR model may not accurately capture the true response curve exhibited by subjects, our experimental design and protocol made it difficult to detect SR by human judging, or even that our results are due to inherent variation. Outcomes that failed to follow the pattern of the characteristic SR response curve were also recently observed in postural sway control (Smith et al., 2020).

Cross-modal improvement in visual thresholds is consistent with the findings of Lugo et al. (2008) and Manjarrez et al. (2007), though with a different white noise stimulation modality. Crucially, we have provided further evidence that cross-modal improvements may exist in human sensory perceptual thresholds. We have expanded upon previous research efforts (Manjarrez et al., 2007; Lugo et al., 2008) with auditory white noise stimulation in both a new modality (vestibular stimulation) and in a more rigorous manner. Through the re-measurement procedure, we ensured independent samples on which to run a statistical test. We suggest this adds a level of rigor, compared to previous studies, which either did not perform any statistical test (Lugo et al., 2008) or a re-measurement and thus producing sampling bias in the “best” threshold measurement (Manjarrez et al., 2007).

Cross-modal improvement specifically in a visual task with the addition of GVS is also consistent with Smith et al. (2020), though that study used a pulsed GVS signal (as opposed to nGVS) and a visual memory search task (as opposed to visual contrast thresholds). Nonetheless, our findings further highlight the potential interaction between applying GVS and visual processing.

We found a correlation between baseline (sham) threshold and improvement. Specifically, we found that those with worse visual contrast thresholds stood to benefit the most from vestibular stimulation. Galvan-Garza et al. (2018) found a similar relationship for in-channel vestibular roll tilt perceptual thresholds. If individuals with innately higher thresholds are the most susceptible for enhancement, there may be benefits of GVS white noise for patient populations. It should be noted that the correlation between sham thresholds and improvement with bnGVS are not independent (since the threshold improvement is the difference between the measured sham threshold itself and the measured threshold with bnGVS). While this is unlikely to have produced a strong correlation when no underlying relationship exists, the false positive rate may be elevated above α = 0.05 in this and previous (Galvan-Garza et al., 2018; Inukai et al., 2018; Keywan et al., 2019) types of correlation analyses.

While we found GVS white noise improved visual thresholds, it did not significantly change auditory thresholds. The mechanisms for in-channel improvements likely differ from those of cross-modal improvements. It seems likely that in both cases there must be resonance in the modality of the sensory perceptual processing. Of course, when white noise stimulation benefits are not observed (such as the auditory threshold arm of our study), there are potentially numerous speculative explanations. It may be that a different auditory tone duration (other than 0.5 s) would be more conducive to cross-modal improvements. Although (Zeng et al., 2000) found in-channel auditory SR at 1 kHz, it is possible that the same 1 kHz frequency might not be conducive to cross-modal improvements or SR. Alternatively, a different range of GVS white noise levels, profile, or application procedure may be necessary. Finally, it may simply be that in our sample of nine subjects, some had lower sham thresholds for other reasons (e.g., fatigue, attention, learning, after-effects). Further research is needed to determine if indeed GVS white noise is ineffective at producing benefits in auditory perception. With only our current results, one must conclude the null hypothesis that nGVS is ineffective at improved auditory thresholds.

We hypothesized that the observed benefit of nGVS on visual perceptual thresholds is due to cross-modal SR. However, it is possible that the application of nGVS enhanced attention or arousal compared to the sham condition. Heightened arousal from suprathreshold GVS could enhance visual thresholds, though we speculate this is unlikely for two reasons. First, while our experiment did not formally inquire at what nGVS level it became perceivable, a previous study (Galvan-Garza et al., 2018) and pilot data suggest amplitudes of less than 0.8 mA are typically undetectable (via either tingling on the skin or illusory self-motion). As seen in Figure 4, the majority of subjects happened to have bnGVS levels <0.8 mA, such that it did not provide a conscious cue to enhance arousal. Second, in a previous study (Lugo et al., 2008) auditory white noise appeared to improve visual thresholds, while a non-noisy 3D-like auditory stimulus did not. The lack of benefit in this control condition designed to heighten arousal, without using white noise to produce resonance, suggests these cross-modal benefits are primarily due to SR. Future studies should uses a similar control condition with GVS, such as a DC or sinusoidal current profile.

We were not able to identify a single GVS level or band of stimulation levels that contain the best stimulation level for all or even most subjects in this study. There is limited data for cross-modal SR, as to whether a single white noise level (or small range of SR noise levels) can be used to enhance all (or most) subject’s perceptual thresholds. For in channel SR, Galvan-Garza et al. (2018) found vestibular perceptual roll tilt thresholds were significantly improved across all subjects at 0.3 and 0.5 mA (but not at 0.2 and 0.7 mA, the other levels assessed), suggesting some amount of consistency in each subject’s best nGVS level. Alternatively, Keywan et al. (2018) found the best nGVS level varied between individuals fairly substantially (0.05–0.3 mA, mean = 0.135 ± 0.86 mA, when testing at 0.05, 0.1, 0.15, 0.2, 0.3, 0.4, and 0.5 mA), as identified using a balance task. It should be noted that our study and these other two studies used slightly different protocols for applying nGVS, such that amplitudes should not be compared directly across studies. Instead, we conclude that while in channel vestibular SR may benefit most subjects using a single nGVS level (Galvan-Garza et al., 2018), for the cross-modal benefits to visual perception we observed (which may not be due to SR) it is critical to identify subject-specific best nGVS levels.

We have not yet shown that the improvement is consistent with existing SR models, as has been shown for in channel vestibular stimulation (Galvan-Garza et al., 2018). Higher plot classification accuracy has potential to generate more conclusive results with respect to SR identification. Notably, when judge #1 performed with very high accuracy while classifying auditory task data (Figure 6C), it became much easier to identify differences between the subject pool and simulated conditions. We speculate that more accurate and objective plot classification may be possible with algorithmic classification (instead of using human judges). Additionally, it is possible that a different underlying curve would be more representative of threshold change with varying GVS. In particular, judge #1 on the auditory task classified subjects differently from both groups of simulations. This indicates that perhaps the subject group did experience some change in auditory thresholds with nGVS but that the underlying model we used was not appropriate. We also note that our statistical tests were run with an α = 0.05 and that the β error (erroneously rejecting the alternative hypothesis) may be higher.

Our study was scoped to identify cross-modal benefits of nGVS, but was not scoped to investigate potential mechanisms, so instead we briefly speculate how nGVS could improve visual thresholds. Multisensory neurons have been shown to exist in both animals and humans (Alex Meredith and Stein, 1986; Frassinetti et al., 2002; Stein and Stanford, 2008) and cross-modal SR is thought to use them (Lugo et al., 2008). There are currently several models for how multisensory information is processed (Seilheimer et al., 2014). Some models use a linear combination of cues (Ohshiro et al., 2011; Fetsch et al., 2012), while others use probabilistic inference (Ma et al., 2006; Shams and Beierholm, 2010) based on reliability of each sensory cue. One study that examined cross-modal SR in the auditory channel with tactile noise hypothesized that the occurrence of cross-modal SR in that modality may be due to the dorsal cochlear nucleus which combines both auditory and somatosensory cues (Krauss et al., 2018). Visual-vestibular integration is required for self-orientation perception (Karmali et al., 2014), with neurons in the dorsal medial superior temporal area capturing this multi-modal sensory integration (Fetsch et al., 2012). One recent study showed cross-modal improvement in a visual memory task with a sub sensory direct current GVS signal (Smith et al., 2020), providing evidence for the hypothesis that visual and vestibular neural pathways are interconnected. It remains an open question whether noisy visual stimulation can reciprocally enhance vestibular perceptual thresholds.

There is evidence that the brain can integrate vestibular and auditory cues to perceive self-motion (Shayman et al., 2020), at least when auditory orientation cues are available. It may be this is a less pronounced sensory integration pathway, such that cross-modal improvements in auditory thresholds are limited with nGVS. While this study was not designed to investigate this potential mechanism, based on current models of multisensory perception, two sensory cues occurring at the same time (e.g., visual stimulus and nGVS) in integrated sensory channels (such as visual and vestibular) may be important for the mechanism of cross-modal SR improving perception of the stimulus.

## Conclusion

We conclude that galvanic vestibular white noise stimulation results in cross-modal improvements in the visual channel, though the response pattern is not necessarily consistent with the current SR model. We found a correlation between subjects’ sham threshold and their improvement magnitude. Further analysis is necessary to identify the scientific mechanism behind the cross-modal improvement and to appropriately model the reduction in perceptual thresholds.

Auditory thresholds appear similar with and without vestibular white noise stimulation. Should improvement in auditory thresholds exist with vestibular white noise stimulation, the improvement may not be large enough to be captured by our study size or threshold measurement precision.

These findings provide further evidence for white noise stimulation may be an effective countermeasure to performance degradation. White noise stimulation may be especially beneficial in operational scenarios where the detection of near threshold stimuli can be critical, such as for astronauts, firefighters, or military personnel. While we only tested normal, healthy individuals, white noise may provide patients with impaired perception a treatment option.

## Data Availability Statement

The raw data supporting the conclusions of this article will be made available by the authors, without undue reservation.

## Ethics Statement

The studies involving human participants were reviewed and approved by The Institutional Review Board at the University of Colorado Boulder. The patients/participants provided their written informed consent to participate in this study.

## Author Contributions

JV: experimental design, data collection, data analysis, and manuscript writing. SS and RR: experimental design, data collection, data analysis, and edit manuscript. AK and PS: data collection, data analysis, and edit manuscript. AA: mentoring professor, experimental design, data analysis, and manuscript writing. TC: mentoring professor, advisor to JV, experimental design, data analysis, and manuscript writing. All authors contributed to the article and approved the submitted version.

## Conflict of Interest

The authors declare that the research was conducted in the absence of any commercial or financial relationships that could be construed as a potential conflict of interest.
